# Coping with early stage breast cancer: examining the influence of personality traits and interpersonal closeness

**DOI:** 10.3389/fpsyg.2015.00088

**Published:** 2015-02-05

**Authors:** Emanuela Saita, Chiara Acquati, Karen Kayser

**Affiliations:** ^1^Department of Psychology, Catholic University of MilanMilan, Italy; ^2^Raymond A. Kent School of Social Work, University of LouisvilleLouisville, KY, USA

**Keywords:** coping strategies, breast cancer, personality traits, close relationships, interpersonal closeness

## Abstract

The study examines the influence of personality traits and close relationships on the coping style of women with breast cancer. A sample of 72 Italian patients receiving treatment for early stage breast cancer was recruited. Participants completed questionnaires measuring personality traits (Interpersonal Adaptation Questionnaire), interpersonal closeness (Inclusion of the Other in the Self Scale), and adjustment to cancer (Mini-Mental Adjustment to Cancer Scale). We hypothesized that diverse personality traits and degrees of closeness contribute to determine the coping styles shown by participants. Multiple regression analyses were conducted for each of the five coping styles (Helplessness/Hopelessness, Anxious Preoccupation, Avoidance, Fatalism, and Fighting Spirit) using personality traits and interpersonal closeness variables (Strength of Support Relations, and Number of Support Relations) as predictors. Women who rated high on assertiveness and social anxiety were more likely to utilize active coping strategies (Fighting Spirit). Perceived strength of relationships was predictive of using an active coping style while the number of supportive relationships did not correlate with any of the coping styles. Implications for assessment of breast cancer patients at risk for negative adaptation to the illness and the development of psychosocial interventions are discussed.

## INTRODUCTION

A diagnosis of breast cancer along with its treatment can challenge patients to deal with a variety of stressors. Although there is a plethora of studies assessing women’s quality of life and adjustment to cancer, we know very little about personality and interpersonal factors that predict the way in which women cope with cancer. Are there specific personality traits that predispose women to cope more effectively with a cancer diagnosis and treatment? What influence does a woman’s relationship with her primary support person or supportive network have on coping? These are important questions to answer in order to develop and deliver psychosocial services that may effectively help cancer patients.

Research investigating the influence of personality factors on the adaptation of women to breast cancer has found that low levels of psychosocial adjustment are significantly linked to factors such as trait anxiety (Bleiker et al., 2000; Van der Steeg et al., 2007), and neuroticism (Millar et al., 2005). In contrast, high levels of adjustment are significantly related to optimism (Carver et al., 2005; Schou et al., 2005). These personality traits can be stronger predictors of women’s adjustment to breast cancer than the severity of the disease (Bleiker et al., 2000; Carver, 2005). For example, when comparing women with benign breast results and those with breast cancer, trait anxiety was a more significant predictor of quality of life than the medical diagnosis itself (Van der Steeg et al., 2007, 2010).

Personality traits not only affect patients’ overall quality of life but also can affect how patients experience symptoms related to the treatment and how they cope with these symptoms. Breast cancer patients who are more neurotic, less agreeable, or more introverted report more fatigue (Michielsen et al., 2007). Patients who are low on neuroticism and high on extraversion and conscientiousness are more likely to participate in exercise during their treatments (Rhodes et al., 2001). While there is strong evidence that personality affects women’s overall adjustment to breast cancer, less is known about how personality influences their coping efforts, that is, how they manage the stress associated with the diagnosis and treatment. Yet in developing psychosocial interventions, unless we fully understand individual differences in approaching the stress of a serious illness, our efforts to assist patients in their coping will be compromised.

Since coping with cancer does not occur in isolation, the social context and the close relationships of the woman need to be taken into account. Viewing women’s coping from a relational perspective, we suggest that coping abilities are influenced by and developed within their close relationships (Kayser et al., 1999; Naaman et al., 2009; Traa et al., 2015). A large body of research over the past decades has confirmed that women’s perceived social support is a critical factor in their adjustment to cancer (Dunkel-Schetter, 1984; Helgeson and Cohen, 1996; Kayser et al., 1999; Kayser and Sormanti, 2002; Kayser and Scott, 2009; Neuling and Winefield, 1988). Furthermore, post-traumatic growth from the cancer experience is positively related to support from the spouse (Calhoun and Tedeschi, 1998; Weiss, 2002), with emotional support rated as the most helpful form of partner support (Rose, 1990; Manne, 1994; Martin et al., 1994; Blanchard et al., 1995; Manne et al., 2014). Emotional support conveys concern, affection and caring and the willingness to listen to worries and discuss important issues (Lichtman et al., 1988; Sormanti and Kayser, 2000). It can help reduce depression and anxiety, and lead to increased quality of life among women with cancer (Helgeson and Cohen, 1996; Martins Silva et al., 2012). Women who report emotional support behaviors are more likely to experience positive mood (Mesters et al., 1997; Alferi et al., 2001), self-esteem, and good physical and role functioning (Roberts et al., 1994; Mesters et al., 1997; Brady and Helgeson, 1999). While the research strongly supports the relationship between emotional support and women’s coping, less is known about the role emotional closeness and quality of the relationship play in their coping abilities. In this study we conceptualize closeness as including the other in the self (Aron and Aron, 1986; Aron et al., 1991, 1997). In their Self-Expansion Theory, Aron and Aron (1986) affirmed that the individual’s sense of self can be modified and expanded to include others, especially if they are people perceived as close to us (Aron et al., 1991). This concept of closeness is similar to self-in-relation in that it posits that through connection, the individual develops a sense of self (Jordan, 2009). Aron and Aron (1986) emphasized that in close relationships the individual may perceive the self as including resources, perspectives, and characteristics of the other. Furthermore, the inclusion of partner’s characteristics into one’s self creates the condition for caring for each other’s well-being (Tomlinson and Aron, 2013). In this sense, closeness can be then considered an interconnection with the significant other (Aron et al., 1992). To our knowledge, this particular concept of closeness has not been investigated in other studies on coping with breast cancer.

The aim of this paper is to determine how five personality factors and the level of closeness with a primary support person influence the type of coping patterns reported by breast cancer patients. First, we hypothesize that women with personality traits such as impulsiveness, narcissism, worry about social image, and stress in social situations are more likely to have coping styles characterized by hopelessness, anxious preoccupation, fatalism, and avoidance. Second, we hypothesize that women who report a high degree of closeness in their relationships and a large number of support persons will use coping strategies characterized by active coping.

## MATERIALS AND METHODS

### PARTICIPANTS

Participants were 72 women with early stage breast cancer who had surgery (lumpectomy or mastectomy) and had started adjuvant treatment at the time of the interview. They were recruited from one hospital in Milan. Institutional Review Board approval was obtained from the University IRB as coordinating center of the study. Each participant completed the required informed consent form. The inclusion criteria for the study required that the participants were: (1) 18 years old or older, (2) free of dementia symptoms and a psychiatric diagnosis, (3) female, (4) Italian-speaking, and (5) had a diagnosis of early stage breast cancer. In the hospital where the study was conducted, women who are screened and tested for BRCA1 and BRCA2 are assigned to a different protocol and they were not included in our sample.

The average age of the participants was 57.7 years (SD = 11.50). Sixty-seven women were Italian native speakers and five were long-term immigrants. The majority of the women (75%) were married and on average had two children. The remaining were either widowed, never married or divorced. About one-third of the patients had children who were young adults (21–39 years of age), and about 25% of the participants had children who were younger than 20 years of age. Overall, the sample was not highly educated. Twenty-nine participants had attended primary school (40.3%), while 38.9% had a high school degree.

More than half of the sample included women who were housewives (62.5%) and the remaining were employed outside the home or unemployed. About 50% of the women discovered the disease through a routine screening (e.g., mammogram), 38.9% by self-examination, and 8.3% by physical symptoms. In the present sample, 14 women had not started treatment at the time the study was conducted (19.4%), while 50% of the participants were receiving multiple forms of treatment (surgery, chemotherapy, and radiation). As part of the interview women were asked if they have had contacted a psychologist in the past, with the majority indicating that they did not perceive the need for psychological care. **Table 1** presents details about the sample demographics.

**Table 1:** Description of demographic and clinical characteristics.

Variable	Breast cancer (*N* = 72) Frequency	Percentage
**Age in years (mean score)**	57.68 (SD = 11.49)	
**Marital status**
Single	4	5.6
Married	54	75.0
Separated/Divorced	6	8.3
Widowed	8	11.1
**Number of children**
No children	8	11.1
1	22	30.6
2	33	45.8
≥3	9	12.5
**Age children (in years)**
No children	8	11.1
<20	18	25.0
21–39	25	34.7
>40	10	13.9
Mixed ages*	11	15.3
**Education**
Primary school	29	40.3
Middle school	8	11.1
High school	28	38.9
BA or higher	7	9.7
**Profession**
Housewife	45	62.5
Employed	21	29.2
Self-employed	5	6.9
Unemployed/Other	1	1.4
**Modality of illness discovery**
Self-exam	28	38.9
Screening	38	52.8
Symptoms	6	8.3
**Treatment modality (three levels)**
No treatment	14	19.4
Single modality	22	30.6
Multiple modality	36	50.0
**Contact with psychologist**
Yes	14	19.4
No	58	80.6

* This category refers to participants with children from different age groups.

### PROCEDURE

Participants were initially interviewed by a psychologist to determine their eligibility and their interest in participating in a clinical research study that involved a psychosocial intervention with the woman’s primary support person. After a brief interview about the cancer experience, study participants completed three questionnaires measuring personality traits, closeness with their primary support person, and coping strategies.

During the interview, women were asked to identify their primary support person. Most frequently, they identified their husbands (51.4%) or children (33.3%) as the prime sources of support (**Table 2**). Four women indicated friends as key support resources, while others listed family members, siblings, or the health care system. What we coined as “unresponsive caregivers” were persons the patients desired to feel close to and involved in their disease experience but, on the contrary, were perceived as distant and unsupportive. Eighteen percent of the women identified partners as “unresponsive caregivers” and the same percentage identified their parents as “unresponsive.” Thirteen percent reported children, friends and family members as persons from whom they desired more involvement. Only one participant indicated the health care system as unsupportive.

**Table 2:** Description of characteristics of identified and unresponsive caregivers.

Variable	Breast cancer (*N* = 72) Frequency	Percentage
**Identified caregiver**
Spouse/partner	37	51.4
Children	24	33.3
Family members/siblings	2	2.8
Friends	4	5.6
Health care system	2	2.8
Others	1	1.4
No caregiver	2	2.8
**Unresponsive caregiver**
No one	31	43.1
Spouse/partner	13	18.1
Parents	13	18.1
Children	6	8.3
Family members	2	2.8
Friends	5	6.9
Health care system	1	1.4

### MEASURES

#### Personality traits

Based on the conceptual framework of the five-factor model (FFM), the 50-item Interpersonal Adaptation Questionnaire (QAI: Questionario di Adattamento Interpersonale) was used to assess participants’ personality traits. The scale was originally developed by Santo Di Nuovo, who had chosen the most effective items from Anglo-Saxon questionnaires investigating personality traits (Watson and Friend, 1969; Gambrill and Richey, 1975; Lange and Jakubowski, 1976; Bakker et al., 1978; Raskin and Hall, 1979; Emmons, 1981, 1987; Turner et al., 1987). He adapted and translated the questionnaire for an Italian-speaking population (Di Nuovo, 1998) and the resulting instrument was validated with 320 subjects. It has been used in studies predicting quality of life of breast cancer patients but to our knowledge has not been utilized to examine coping strategies used by cancer patients.

Five subscales measure the following personality factors. *Assertiveness* is the ability of a person to show behaviors expressing self-affirmation or assertiveness. This aptitude involves the capacity to affirm one’s own needs and principles which can further enhance one’s interpersonal relationships (Lange and Jakubowski, 1976; Bellack and Hersen, 1979). *Impulsiveness* refers to the uncontrolled expression of feelings and aggressiveness. This kind of hostile behavior tends to become a stable and prevailing strategy for the person to solve conflicts in his/her relationships. Impulsiveness and aggressiveness tend to provoke reactions and consequences from others that can lead to isolation or persistent conflict (Caprara, 1976; Hollandsworth, 1977; Nencini and Belcecchi, 1979; Fehrenbach and Thelen, 1982; Di Maria and Di Nuovo, 1984; Caprara et al., 1991). The subscale of *Narcissism* measures a narcissistic orientation that is characterized by great attention to the self and personal needs. Although an adequate level of self-esteem is essential to maintain interpersonal relationships, a consistent and excessive display of narcissism can hinder interpersonal relationships and can lead to clinical diagnoses such as borderline and other personality disorders (Kohut, 1976; Kernberg, 1980; Millon, 1981). Social anxiety is conceptualized as *Worry about one’s social image*. This subscale focuses on the negative thoughts and worry of the individual in a situation that requires close contact with others. This characteristic has the potential to affect social relationships, primarily by avoiding or losing the motivation for affiliation (Clark and Arkowitz, 1975; Buss, 1980; Glass and Merluzzi, 1981; Villone Betocchi and Asprea, 1994). A final subscale, *Stress in Social Situations*, measures stress that is experienced in social situations that are perceived by the individual as threatening or dangerous. Stress involves a state of tension that can develop from an acute stress to a chronic condition in which the level of stress remains constant and potentially results in harmful long-term effects.

The QAI has shown good discriminant validity and reliability (Di Nuovo, 1998). Analyses with an Italian sample indicated significant differences between males and females: women tended to report higher levels of Assertiveness and Stress in Social Situations while men were more likely to rate higher on Narcissism (Di Nuovo, 1998).

#### Interpersonal closeness

The degree of closeness or connection with the primary support person was measured by the Inclusion of the Other in the Self Scale (IOS; Aron et al., 1992). This instrument can be applied to a variety of interpersonal relationships (Gómez et al., 2011), but it is typically used to assess closeness in romantic relationships (Frost and Forrester, 2013; Tomlinson and Aron, 2013). As mentioned earlier, Aron and Aron (1986) posit that in a close relationship the individual acts as if some or many aspects of the other partner are part of the individual’s sense of self (Aron and Aron, 1986; Aron et al., 1991). In other words, an individual perceives himself or herself as including resources, perspectives, and characteristics of the other. The measure consists of seven pairs of overlapping circles that are drawn to show varying levels of overlap, indicating the degree of closeness in the relationship. The less the circles overlap, the less inclusion exists between the two persons; the more two circles overlap, the more inclusion exists. A 7-point scale is used to score the degree of closeness. In the present study, we asked the individual to indicate up to five persons who provide support to them and describe each relationship by choosing one of the seven circles. Two measures of interpersonal closeness were created: strength and number of support relations. Strength of support relations refers to the sum of the degree of closeness indicated by the subject while the variable of number of support relations refers to the number of people identified in the IOS scale (with a maximum of 5 choices).

#### Styles of coping with cancer

Women’s coping styles were the outcome variable for the study. To identify the prevailing coping style used to cope with cancer, the Mini-Mental Adjustment to Cancer Scale (Mini-MAC; Watson et al., 1994) was selected. The instrument is a 29-item questionnaire resulting from a previous version, the Mental Adjustment to Cancer Scale (Greer and Watson, 1987; Watson and Greer, 1988; Watson et al., 1988, 1989). Coping is conceptualized as cognitive and behavioral responses based on appraisals that reduce the threat of cancer (Lazarus, 1966; Lazarus and Folkman, 1984). The factor structure of the original scale identified the following coping styles: Fighting Spirit, Hopeless/Helplessness, Anxious Preoccupation, Fatalism, and Avoidance. *Hopelessness/Helplessness* indicates a coping style characterized by high levels of anxiety and depression, along with the belief of low control on events and giving-up. Individuals with a *fatalistic* coping style are characterized by low levels of anxiety and depression, low sense of control, resignation, and passive acceptance of fate. *Anxious Preoccupation* is used to describe a coping modality with high levels of anxiety and worry about the cancer diagnosis, which can impact the quality of life of the individual. The patient is either looking for constant reassurance or is distancing herself/himself from the healthcare environment. *Avoidance* indicates the tendency to minimize the event of cancer and to refrain from the search of information. *Fighting Spirit* is characterized by an optimistic attitude toward one’s ability to cope with the illness. Next to low levels of anxiety and depression, individuals characterized by fighting spirit tend to perceive the illness as a challenge. They present diverse and flexible cognitive strategies, which contribute to a positive appraisal of the experience. This coping style has been associated to better psychological morbidity, increased sense of control and better prognosis (Pettingale et al., 1985; Burgess et al., 1988).

The Italian version of the Mini-MAC was validated by Grassi and Watson (1992) and Grassi et al. (2005). The version was obtained by translating and back translating the scale from the original language by Italian doctors fluent in English. The original factor structure was used to obtain scores on the five sub-scales identified in previous research. The Cronbach’s Alpha coefficients were similar to the original study (Watson et al., 1994) and the construct validity of the instrument was confirmed (Grassi et al., 2005). The Italian adaptation revealed good reliability for Anxious Preoccupation, Hopelessness and Cognitive Avoidance, while Fighting Spirit and Fatalism were found to be more inconsistent. A factor analysis identified the same five factors of the original version, with minor discrepancies in the factor loading and their components. Hence, the Italian version of Mini-MAC appears to be an acceptable instrument to investigate cancer patients’ coping styles. Grassi et al. (2005) emphasize that, from a clinical perspective, Hopelessness, and Anxious Preoccupation appear to be the most significant indicators of psychological distress and maladjustment.

### DATA ANALYSIS

Bivariate analyses were conducted with all of the variables to check for multicollinearity. A series of regression analyses was conducted to test the influence of the personality traits and interpersonal closeness on coping styles.

## RESULTS

### CORRELATIONS AMONG PERSONALITY TRAITS, INTERPERSONAL CLOSENESS, AND COPING STYLES

Bivariate analysis revealed several significant correlations (**Table 3**) between the five subscales of the QAI, the Mini-MAC and measures of interpersonal closeness. Narcissism was positively correlated with Impulsiveness (*r* = 0.26, *p* < 0.05), Stress in Social Situations (*r* = 0.24, *p* < 0.05), and Social Image (*r* = 0.45, *p* < 0.001). Impulsiveness was associated Stress in Social Situations (*r* = 0.24, *p* < 0.05), while Social Image was highly correlated with Stress in Social Situations (*r* = 0.52, *p* < 0.001). Among the Mini-MAC subscales, Helplessness/Hopelessness was positively correlated with Anxious Preoccupation (*r* = 0.39, *p* < 0.01) and Avoidance (*r* = 0.25, *p* < 0.05) and negatively correlated with Fighting Spirit (*r* = –0.25, *p* < 0.05). Anxious Preoccupation was positively correlated with Avoidance (*r* = 0.32, *p* < 0.01) and Strength of Support Relations (*r* = 0.25, *p* < 0.05). Fighting Spirit was correlated with Assertiveness (*r* = 0.37, *p* < 0.01) and Strength of Support Relations (*r* = 0.35, *p* < 0.01). A negative correlation was registered for Strength of Support Relations with Impulsiveness (*r* = –0.33, *p* < 0.01). Finally, a negative correlation was registered between Number of Support Relations and Impulsiveness (*r* = –0.24, *p* < 0.05) and a positive correlation with Strength of Support Relations (*r* = 0.87, *p* < 0.001). No significant correlations were identified between demographic and clinical variables and the selected outcome measures.

**Table 3:** Correlations among personality traits, interpersonal closeness, and coping styles variables.

Correlations	1	2	3	4	5	6	7	8	9	10	11	12
Helplessness/hopelessness	–	0.39**	0.25*	-0.09	-0.25*	-0.09	0.15	-0.08	-0.01	0.09	-0.10	-0.22
Anxious preoccupation	–	–	0.32**	0.15	0.08	0.09	-0.13	-0.14	0.23	0.12	0.25*	0.20
Avoidance	–	–	–	0.14	0.04	0.23	-0.04	0.11	0.17	0.13	-0.002	-0.17
Fatalism	–	–	–	–	0.17	0.17	-0.17	0.03	-0.06	0.18	0.05	0.01
Fighting spirit	–	–	–	–	–	0.37**	0.01	-0.02	0.08	0.23	0.35**	0.20
Assertiveness	–	–	–	–	–	–	0.10	0.05	0.10	0.05	0.16	0.004
Narcissism	–	–	–	–	–	–	–	0.26*	0.45***	0.24*	-0.15	-0.09
Impulsiveness	–	–	–	–	–	–	–	–	0.06	0.24*	-0.33**	-0.24*
Social image	–	–	–	–	–	–	–	–	–	0.52***	0.08	0.09
Stress in social situations	–	–	–	–	–	–	–	–	–	–	-0.014	-0.02
Strength of support relations	–	–	–	–	–	–	–	–	–	–	–	0.87***
Number of support relations												–

*p < 0.05; **p < 0.01; *** p < 0.001. Bold values are significant correlations.

### PERSONALITY TRAITS AS PREDICTORS OF COPING STYLES

Using multiple regression analysis, we analyzed personality traits as possible predictors of each coping style. Fighting Spirit was the only coping style that was significantly influenced by personality traits. The analysis revealed that women characterized by Assertiveness (β = 0.38, *p* < 0.01) and Stress in Social Situations (β = 0.32, *p* < 0.05) tended to report higher scores on Fighting Spirit (**Table 4**).

**Table 4:** Multiple regression analysis for Fighting Spirit predicted by personality traits.

		Fighting Spirit			
Variables	*B*	Standard error(B)	β	*t*	Part correlation
Constant	1.94	0.39		4.98***	
**Assertiveness**	**0.85**	**0.25**	**0.38**	3.35**	**0.38**
Impulsiveness	-0.17	0.19	-0.110	-0.90	-0.10
Narcissism	-0.04	0.25	-0.02	-0.15	-0.02
Social image	-0.21	0.25	-0.13	-0.85	-0.10
**Stress in social situations**	**0.54**	**0.24**	**0.32**	2.28*	**0.26**

Overall *R*^2^ = 0.204, Adjusted *R*^2^ = 0.140, *F*(5,62) = 3.19, *p* = 0.013. *p < 0.05; **p < 0.01; ***p < 0.001.

### INTERPERSONAL CLOSENESS AS A PREDICTOR OF COPING STYLES

We used two measures of the interpersonal closeness between the patient and her primary support person: the degree of closeness as measured by the IOS scale and the number of support persons reported by the participant. Given the high correlation registered among the two variables and the associated multicollinearity, simple linear regressions have been conducted. As no significant results were obtained including number of support persons as predictor, in line with the original contribution authors present here only significant results for strength of the supportive relationships.

Simple linear regressions were conducted for each coping styles. Strength of Support Relations was a significant predictor of Fighting Spirit (β = 0.35, *p* < 0.01) and Anxious Preoccupation (β = 0.25, *p* < 0.01) coping styles in the present sample (**Tables 5** and **6**).

**Table 5:** Simple linear regression for Fighting Spirit predicted by interpersonal closeness.

		Fighting Spirit		
Variables	*B*	Standard error(B)	β	*t*
Constant	2.88*	0.14*		20.81***
**Strenght of support relations**	0.02*	0.01*	0.35*	3.02**

Overall *R*^2^ = 0.120, Adjusted *R*^2^ = 0.107, *F*(1,67) = 9.14, *p* = 0.004. *p < 0.05; **p < 0.01; ***p < 0.001.

**Table 6:** Simple linear regression for Anxious Preoccupation predicted by interpersonal closeness.

		Anxious preoccupation		
Variables	*B*	Standard error(B)	β	*t*
Constant	2.04*	0.17*		11.7***
**Strenght of support relations**	0.02*	0.01*	0.25*	2.20**

Overall *R*^2^ = 0.065, Adjusted *R*^2^ = 0.051, *F*(1,70) = 4.84, *p* < 0.05. *p < 0.05; **p < 0.01; ***p < 0.001.

## DISCUSSION

The aim of this study was to examine the influence that personality traits and perceived closeness with a primary support person have on women’s abilities to cope with stresses related to breast cancer. The present findings indicate that patients who have personality traits of assertiveness and social anxiety tend to use coping strategies characteristic of Fighting Spirit. This suggests the tendency to use an active form of coping when feeling a level of anxiety and/or the ability to be assertive in stressful situations. This highlights a coping pattern of women who appraise the disease as a challenge or a threat which mobilizes their assertiveness.

Our focus on the relational aspects of women’s coping involved the notion of closeness (Aron and Aron, 1986; Aron et al., 1991). By referring to the IOS Scale we created two variables: the strength and the number of the support relations. In our sample the Strength of the Support Relations variable was predictive of Anxious Preoccupation and Fighting Spirit coping styles. This finding may appear to be counterintuitive. However, a breast cancer patient who feels very close to her partner may be preoccupied with her partner’s anxiety about the cancer and attempting to cope with the cancer-related stress on her own as a way to buffer or protect her partner from emotional distress. The reciprocal influence partners have on each other has been documented by previous studies (Hagedoorn et al., 2008; Northouse and McCorkle, 2010). In particular, the literature on dyadic coping reveals how the ability of the dyad to identify the diagnosis as a stressor that affects both of them and to activate new coping strategies leads to higher marital satisfaction and psychological and physical well-being (Bodenmann, 2000; Walen and Lachman, 2000; Dehle et al., 2001; Badr, 2004; Traa et al., 2015). Some studies have found that patients in close relationships can worry a great deal about the effects of their disease on loved ones. Hence, the request of support can be a delicate thing. While they hope they can lean on the loved ones in times of need, they may also simultaneously try to protect them from emotional pain and sorrow resulting from their cancer experience, and to support them (D’Errico et al., 1999; Kayser and Scott, 2008; Saita and Cigoli, 2009). The consequence of this ambivalence can be observed in the high level of anxiety shown by the women. In order to protect the loved ones from such traumatic and painful experience, patients tend to minimize the illness. They show indifference and do not search for further information or details about treatments and the prognosis of cancer. In contrast, there are women who find the resources to face and to cope with the illness relying on their loved ones.

It is also interesting to report that studies on resiliency confirm the key role of the supportive system when the individual is confronted with potentially traumatic events. For example Galatzer-Levy et al. (2012) found that social network size and social integration have significant roles on the amount of distress experienced by students transitioning to graduate life. Similar to our results, some studies have also identified that it is not the quantity but rather the quality of the relationships established within the social network to promote adaptation (Morosanu et al., 2010; Galatzer-Levy et al., 2012). Since support within the context of a close relationship leads to better outcomes, we propose that forms of interventions that focus on the dyad of patient and primary support person would be appropriate and potentially effective in promoting and enhancing quality of life for both the patient and partner during treatment for breast cancer (Kayser and Scott, 2008; Saita et al., 2014).

We note some limitations to our research. First, the sample was quite homogenous in terms of ethnicity and socio-economic status and does not allow us to explore cultural differences (Kayser et al., 2014). Second, the design of the study was cross-sectional and longitudinal data could have provided a better understanding of the stability of the women’s coping styles. Also it would have allowed us to explore the interaction between personality characteristics and different levels of stress experienced throughout the cancer journey. Finally, the sample size affected the selection of predictors for the regression analyses (Tabachnick and Fidell, 2007) and our ability to assess the conjoint effect of personality traits and close relationships on women’s adjustment to cancer. Although limitations are present, the study can be considered a starting point for additional investigations. By including larger samples, future studies will be able to meet the requirements for higher levels of analysis. Furthermore, the focus of future contributions can be on the types of psychosocial interventions that may be effective with patients with different personality traits. In particular, it will be important to explore if some patients benefit from an individual psychosocial intervention more than a group or couple-based intervention.

This study examined the influence of personality traits and close relationships on the coping style of women with breast cancer. Results revealed how personality traits contribute to the coping process, but also the importance of close relationships. The ability to adjust to the cancer experience is impacted not only by medical treatments, but also by relational and intrapsychic characteristics of the individual (Saita and Cigoli, 2009). When developing psychosocial interventions for cancer patients, it is therefore essential to consider the relevance of significant relationships the patient establishes with partners, friends and healthcare professionals. It is within the context of these interpersonal relationships that optimal emotional adjustment to cancer can be addressed.

## Conflict of Interest Statement

The authors declare that the research was conducted in the absence of any commercial or financial relationships that could be construed as a potential conflict of interest.
